# Supplementary material for: Impact of Clinical Use of Probiotics on Preterm-Related Outcomes in Infants with Extremely Low Birth Weight
Source: Nutrients. 2024 Sep 5;16(17):2995. doi: 10.3390/nu16172995 (PMC11397160; doi:10.3390/nu16172995)
Supplement: Supplementary file 1 [file nutrients-16-02995-s001.zip › nutrients-3140982-supplementary.pdf]

Impact of Clinical Use of Probiotics on Preterm-Related Outcomes in Infants with Extremely Low Birth Weight

Wei-Hung Wu, Ming-Chou Chiang, Ren-Huei Fu, Mei-Yin Lai, I-Hsyuan Wu, Reyin Lien and Chien-Chung Lee *

Table S1. a nutritional table for the two probiotic formulations used in this study.

	MoProbi-LR 150mg	Infloran 250mg
**Ingredient**	*Lactobacillus rhamnosus GG* ATCC53103 $10x10^8$ cfu/g	*Lactobacillus acidophilus* $150x10^9$ cfu/g and *Bifidobacterium bifidum* $150x10^9$ cfu/g
